# Prefrontal cortex activation while walking did not change but gait speed improved after a randomized physical therapy intervention

**DOI:** 10.1007/s40520-023-02666-7

**Published:** 2024-02-17

**Authors:** Andrea L. Rosso, Emma M. Baillargeon, Subashan Perera, Jessie VanSwearingen, Caterina Rosano, Theodore J. Huppert, Jennifer S. Brach

**Affiliations:** 1 Department of Epidemiology, University of Pittsburgh, Pittsburgh, PA USA; 2 Division of Geriatric Medicine, University of Pittsburgh, Pittsburgh, PA USA; 3 Department of Physical Therapy, University of Pittsburgh, Pittsburgh, PA USA; 4 Department of Electrical and Computer Engineering, University of Pittsburgh, Pittsburgh, PA USA

**Keywords:** Gait, Aging, Dual-task, Prefrontal cortex, Functional near-infrared spectroscopy, Physical therapy, Clinical trial

## Abstract

**Background:**

Higher prefrontal cortex (PFC) activation while walking may indicate reduced gait automaticity.

**Aim:**

We examine whether PFC activation during walking improves after training in older adults at risk for mobility disability.

**Methods:**

Forty-two adults aged ≥ 65 participated in a randomized clinical trial (NCT026637780) of a 12-week timing and coordination physical therapy intervention to improve walking (*n* = 20 intervention, *n* = 22 active control). PFC activation was measured by functional near-infrared spectroscopy (fNIRS) during four walking tasks over 15 m, each repeated 4 times: even surface walking, uneven surface walking, even dual-task, uneven dual-task; dual-task was reciting every other letter of the alphabet while walking. Gait speed and rate of correct letter generation were recorded. Linear mixed models tested between arm differences in change of fNIRS, gait speed, and letter generation from baseline to follow-up (12-week, 24-week, and 36-week).

**Results:**

Intervention arms were similar in mean age (74.3 vs. 77.0) and baseline gait speed (0.96 vs. 0.93 m/s). Of 24 comparisons of between arm differences in the fNIRS signals, only two were significant which were not supported by differences at other follow-up times or on other tasks. Gait speed, particularly during dual-task conditions, and correct letter generation did improve post-intervention but improvements did not differ by arm.

**Discussion and Conclusions:**

After training, PFC activation during walking generally did not improve and did not differ by intervention arm. Improvements in gait speed without increased PFC activation may point toward more efficient neural control of walking.

**Supplementary Information:**

The online version contains supplementary material available at 10.1007/s40520-023-02666-7.

## Introduction

Mobility limitations affect one-quarter to one-half of community-dwelling older adults [1, 2] with an associated $42 billion in annual health care costs [3]. Current exercise and therapy recommendations for older adults typically target the musculoskeletal and cardiopulmonary systems and overlook the brain’s role in mobility. In response, we developed a motor skill training approach using goal-oriented, task-specific timing, and coordination exercises [4, 5]. The goal of this motor skill training is to challenge the brain to adapt and relearn the sequence of movements and timing with the postures and phases of gait to improve walking. Thereby, improvements in walking may occur by restoring the pattern of brain and neuromuscular activation that optimizes the use of physiologic capacity to meet the demands of walking [5].

The automaticity of walking decreases with age, leading to a greater dependence on attention for motor control [6]. The timing and coordination training is a task-oriented intervention with the goal of returning the older adult to a skilled “expert” walker; in other words, restoring more automatic motor control during walking. The extent of automatic vs. attentional motor control can be detected through functional near-infrared spectroscopy (fNIRS) of the PFC during complex walking tasks [7]. Attentional motor control relies on prefrontal–parietal pathways; with training, motor control shifts toward striatal–cerebellar pathways [8]. Walking in healthy adults is an automatic process with little reliance on the PFC. However, as automatic motor control diminishes in older adults due to impairments in the brain and other systems [5, 7], activation of the PFC during walking tasks increases, particularly under challenging walking conditions [9, 10]. The left dorsolateral PFC (dlPFC) is thought to be particularly relevant for challenging walking conditions in older adults [11, 12].

Current evidence for the plasticity of PFC involvement in walking is limited. A motor learning dance video game intervention resulted in a decrease in PFC activation during treadmill walking in older adults as they became more trained, resulting in a more “youthful” fNIRS signature [13]. Transcranial direct stimulation of the PFC in older adults may also improve walking performance, particularly during challenging walking conditions [14, 15]. Studies in individuals with Parkinson’s disease also demonstrate that behavioral interventions can lead to more automatic motor control as evidenced by lower PFC activation after the intervention [16–18].

Here, we examine the effects of a 12-week timing and coordination training program intended to improve walking in older adults at risk for mobility disability compared to a standard physical therapy intervention on PFC activation. The primary results of this randomized clinical trial found that there were clinically meaningful improvements in gait speed but that improvements were no greater in the timing and coordination training compared to the standard physical therapy arm [19]. We proposed that the timing and coordination training would improve automaticity of motor control during walking, resulting in decreased PFC activation by fNIRS during challenged walking, compared to usual care. We further hypothesized that these differences would be driven by changes in activation specifically in the left dorsolateral PFC (dlPFC) and that the intervention would result in better performance defined by faster gait speed and better cognitive performance during dual-task walking. Differential changes in PFC activation could occur in the absence of between arm changes in gait speed, indicating improved automaticity and more efficient walking with the timing and coordination training.

## Methods

We follow the CONSORT reporting guidelines [20].

### PRIMA study

The Program to Improve Mobility in Aging (PRIMA) is described in detail elsewhere [4] (NCT02663778; https://clinicaltrials.gov/ct2/show/NCT02663778). PRIMA was a randomized clinical trial of motor skill training to improve timing and coordination of gait. The primary outcome was gait speed, which improved significantly but improvements did not differ by intervention arm [19]. The parent study enrolled 249 participants who were randomized to either standard physical therapy or to standard-plus timing and coordination training, referred to as standard and standard-plus below. Participants were recruited from the greater Pittsburgh, USA area using the Pittsburgh Claude D. Pepper Older Americans Independence Center Research Registry. Inclusion criteria included age 65 years or older and walking speed between 0.6 and 1.2 m/s, indicating increased risk for negative outcomes but ability to participate in the intervention [21]. Persons who were unable to participate in testing had medical conditions which would make participation unsafe, or who had plans to leave the area during the study were excluded (for detailed exclusion criteria [4]). Recruitment flow of the main trial and the randomization process are detailed elsewhere [19].

This study complies with standards of the Declaration of Helsinki. The University of Pittsburgh Institutional Review Board approved the protocol and all participants provided written informed consent prior to participation.

### PRIMA-NIRS ancillary study

Recruitment for the PRIMA-NIRS ancillary study began after the parent study with the goal to recruit at least 50 participants. Recruitment started in 2018, paused in March 2020 due to COVID-19 restrictions, and ended without restart in September 2020. Follow-up visits were conducted in parallel with the parent study at 12 (immediately post-intervention), 24, and 36 weeks. The first seven participants were enrolled as part of a pilot feasibility study and were only invited to return for PRIMA-NIRS visits at 12 and 24 weeks.

### Interventions

Both intervention arms included 2 clinic visits/week for 12 weeks. Intervention arms included a standard physical therapy and a standard-plus timing and coordination training. Intervention protocols are described briefly here; details are reported elsewhere [4].

*Standard*: The standard intervention included progressive lower extremity strength training and endurance training. Strength training included hip extensor and hip abductor muscle strengthening, plus strengthening of any 2 other lower extremity muscle groups based on individual needs. The endurance training consisted of treadmill walking at a submaximal workload with a self-reported rate of perceived exertion (RPE) of 10–13, somewhat hard.

*Standard Plus*: The active intervention arm included the standard intervention plus timing and coordination training. Time spent in the standard intervention components was reduced to keep total intervention time equal between groups. The timing and coordination program used goal-oriented, progressively more difficult stepping and walking patterns to promote proper timing and coordination of stepping integrated with the phases of the gait cycle [22]. The goal was to improve the motor skill of walking by realigning biomechanical and neuromotor programs and improving feedback for adjusting movements.

*Behavioral:* Both intervention groups received a physical activity behavioral change intervention based on the Group Lifestyle Balance™ program, [23] totaling 16 sessions.

### Data collection

All data were collected by assessors who were blind to intervention arm.

#### Challenged walking protocol

Participants were asked to complete both cognitive and mobility tasks along a track with 15 m straightaways (for detailed description see [24]). The mobility tasks consisted of four combinations of two surfaces, even and uneven, and two task conditions, single- and dual-task. Participants completed four repetitions of each of the four combinations of surfaces and tasks in pseudo-randomized order to ensure that walking always progressed continuously around the track, alternating even and uneven surfaces. The even surface consisted of level flooring without obstacles or perturbations. The uneven surface consisted of 1.5 cm high wood prisms arranged randomly at a density of 26 pieces/m^2^ underneath carpeting [25]. A 20 s quiet standing period was included before each task to act as the baseline condition for fNIRS recordings. The single task involved the participant walking at their comfortable walking pace while not speaking. The dual-task required participants to recite every other letter of the alphabet out loud starting with the letter “B” [26] while walking. Participants were instructed to return to the beginning of the alphabet and continue if they reached the end of the alphabet before completing the walk.

Time to walk, 15 m segments, was recorded by stopwatch and converted to gait speed in m/s. The rate of correct letter generation was calculated as the number of correct letters voiced divided by the number of seconds during each task period (i.e., time to walk the m segment).

#### Functional near-infrared spectroscopy

**Fig. 1:**
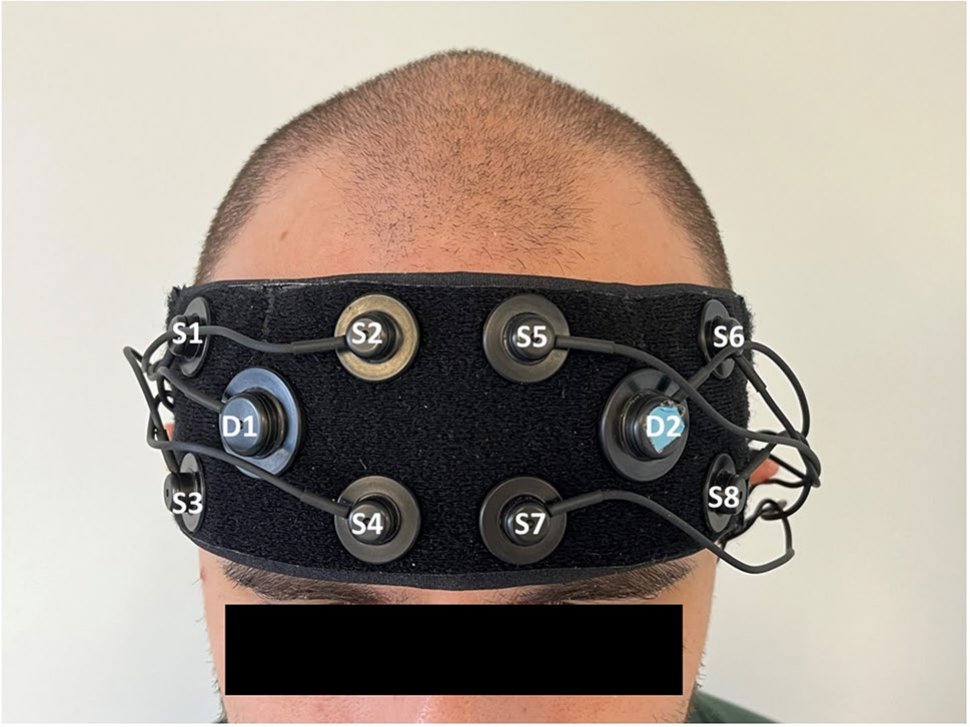
Location of the OctaMon functional near-infrared spectroscopy (fNIRS) probe (Artinis Medical Systems; Netherlands) with 8 sources (1–8) and 2 detectors (D1–D2) covering the forehead of participants

fNIRS data collection, analysis, and reporting are in line with consensus recommendations [27]. Participants wore an eight-channel continuous wave fNIRS headband (Octa-Mon; Artinis Medical Systems; Netherlands) to estimate changes in activity in the right and left prefrontal cortical regions. The fNIRS instrument measured oxygenated (HbO) and deoxygenated (Hbr) hemoglobin concentrations at 850 and 760 nm, respectively, with four sources and one detector on each side of the head. Source to detector distance was 35 mm. For consistent placement, the center of the optodes was aligned with the center of the nose and the bottom of probe was placed just above the eyebrow. The probe covered approximately bilateral Brodmann areas BA9, BA44, BA45, and BA46 (Fig. 1). Optical data were collected at 10 Hz and stored using OxySoft software (Artinis Medical Systems; Netherlands).

#### fNIRS processing

Raw signals recorded by the fNIRS device were exported to Matlab (MATLAB and Statistics Toolbox Release 2021b, The MathWorks, Inc., Natick, MA) and processed using the NIRS Brain AnalyzIR Toolbox [28]. The start time and the end time of each walking task were recorded and used to label each trial of the fNIRS signals. Trials were trimmed to include 2 s of data before and after labeled tasks to reduce noise. Flat channels due to saturation or equipment error were removed from analysis. Visual checks were performed to confirm no time overlap across tasks and for data quality. Observations were excluded when fNIRS or time data were missing or for protocol deviations (e.g., participant walked during a standing task). Excluded observations from any one visit ranged from 1.8 to 8.9% of available data. Because all walking tasks were completed four times, exclusion of some observations did not impact overall data availability or quality.

The fNIRS light intensity signals were converted to optical density and resampled to 4 Hz to reduce computational burden. The modified Beer–Lambert law was applied to calculate concentration of HbO and Hbr (both in µM) across time from optical density data [29]. In first-level modeling, a canonical model with autoregressive pre-whitening approach using iteratively reweighted least-squares (AR-IRLS) was implemented to model changes in HbO and Hbr for each task relative to a global baseline [30]. We conducted channel-wise Student’s t tests comparing the level of HbO change for each task from the quiet standing baseline state. A mixed effects model with fixed effect of tasks was applied to combine the results of the four tests for each visit. The mixed effects model used robust weighted least-square regression with the covariance matrix derived from the first-level model. We obtained a single *t*-statistics for each channel and task per visit. These channel-specific t-statistical values were averaged over the right and left hemispheres of the PFC for all primary analyses. Secondary analyses focused on the left dlPFC using channel-specific values from D2S6 and D2S8 (Fig. 1).

#### Descriptive variables

Demographic characteristics, including age, gender, race, marital status, living arrangement, and educational attainment, were self-reported at screening. The Duke comorbidity index was used to determine whether 8 different physiologic systems were affected by chronic conditions at screening. The Modified Mini-Mental State Exam (3MS) [31] was also administered at screening to assess overall cognitive function. Depressive symptoms were determined by the Geriatric Depression Scale [32]. Speed of processing and switching ability were assessed by Trails A and B tests [8]. Height and weight were measured using standard clinical protocols. Falls in the prior 12 months as well as fear of falling were determined with single-item questions. Gait speed was averaged over six passes of a 14-foot instrumented walkway (Protokinetics LLC, Havertown, PA).

#### Sample size calculation

At the time, this study was proposed, there was limited data from which to conduct accurate power analyses for fNIRS outcomes. In addition, our recruitment was based on a convenience sample from a larger clinical trial and our analyses were considered exploratory. As such, we did not conduct separate power analyses for this research question. However, other published studies have found significant differences in fNIRS measures with as little as 14–19 participants per group [13, 33]. Our primary outcome was differences at the 12-week time period and our number in each intervention arm with data at that time period is within this range.

#### Data analysis

We used independent *t*-samples, chi-square, and Fisher’s exact tests, as appropriate, to compare participant characteristics and baseline measurements between the two groups. We fitted a series of linear mixed models using the SAS® MIXED procedure (SAS Institute, Inc., Cary, NC) with the dependent variable being change from baseline in each of the fNIRS and performance measures. Also included in the models were intervention arm (standard/standard-plus), follow-up time point (at 12/24/36 weeks) and their interaction as fixed effects of interest, baseline value of the outcome as a fixed-effect covariate, and a participant random effect. We used appropriately constructed means contrasts to estimate the difference between intervention-related changes at 12 (immediate effect), 24, and 36 weeks (effect sustained over the longer term).

## Results

**Fig. 2:**
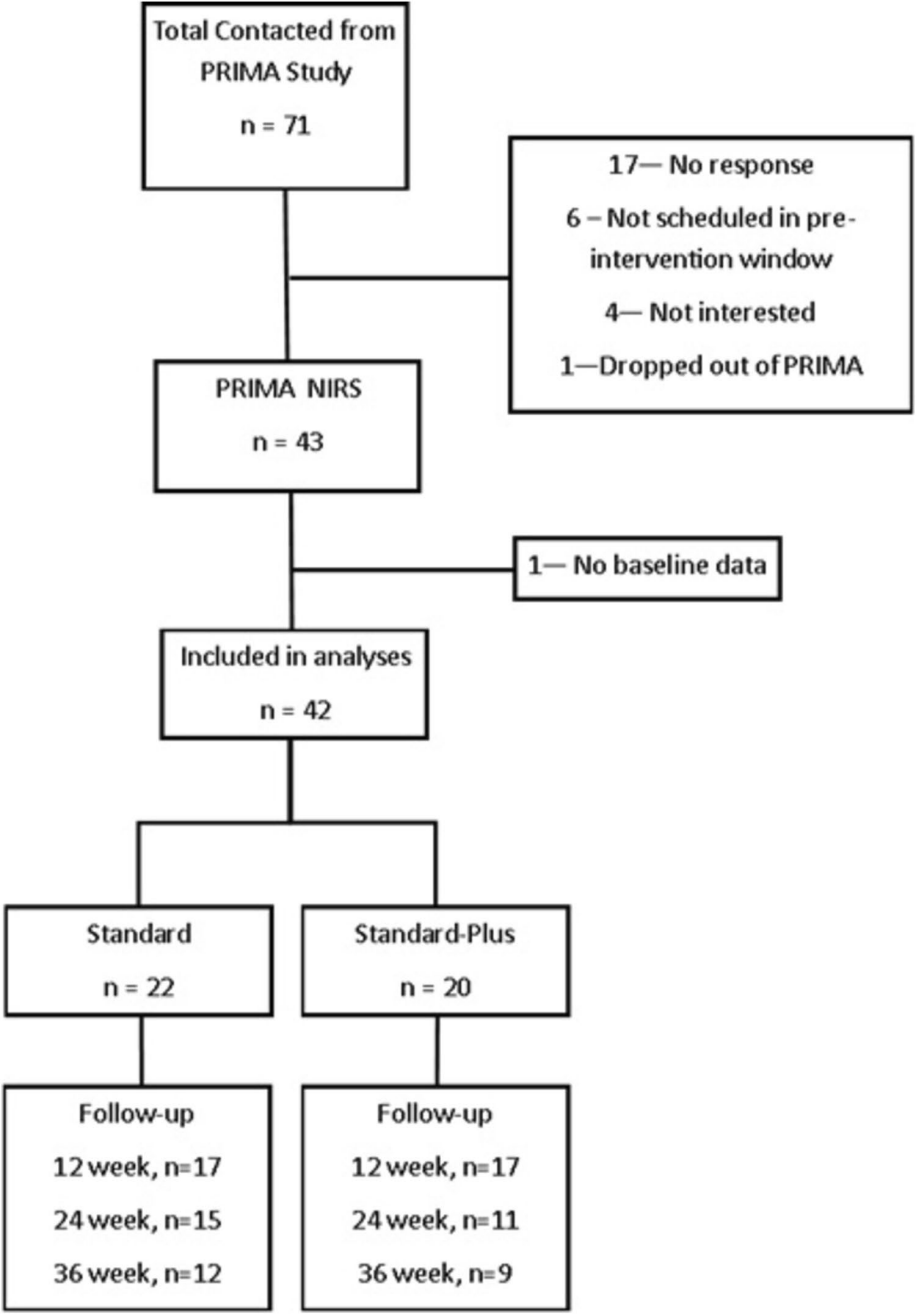
Flow chart of enrollment from the parent randomized control trial into the functional near-infrared spectroscopy ancillary study and follow-up

Seventy-one PRIMA participants were contacted to participate in the PRIMA-NIRS ancillary (Fig. 2). Of those contacted, 17 could not be reached within the designated time window, 6 could not be scheduled within the window, 4 were not interested in participating, and 1 dropped out of the parent study. Forty-three participants were enrolled in the PRIMA-NIRS ancillary study. One enrolled participant did not complete baseline fNIRS testing, resulting in 42 participants who are included in these analyses. Of these, 20 were randomized to the standard-plus arm and 22 to the standard arm (Fig. 2). At the 12-week, post-intervention visit, 17 participants in the standard-plus and 17 participants in the standard arm completed the visit. At 24 weeks, 11 in the standard-plus and 15 in the standard arms completed the visit and at 36 weeks, 9 in the standard-plus, and 12 in the standard arm completed the visit (Fig. 2).

Participants had a mean age in the mid-seventies and were predominantly female and White (Table 1). On average, participants had comorbidities in 3 physiologic systems, low depressive symptoms by the GDS, and performed well on the 3MS (Table 1). The average usual pace gait speed at baseline was slightly below 1.0 m/s in both arms (Table 1).

**Table 1:** Baseline demographic and health characteristics of older adults enrolled in a randomized clinical trial of a timing and coordination physical therapy intervention (*n* = 42)

Characteristic	Standard +	Standard	p-Value
	*N* = 20	*N* = 22	
	Mean (SE) or *n* (%)	Mean (SE) or *n* (%)	
Age	74.3 (7.8)	77.0 (5.0)	0.1
Female gender	14 (70.0%)	11 (50.0%)	0.2
Race			0.7
Black	1 (5.0%)	4 (18.2%)	
White	18 (90.0%)	18 (81.8%)	
Other	1 (5.0%)	0 (0.0%)	
Live alone	9 (45.0%)	8 (36.4%)	0.5
Marital status			0.5
Married	10 (50.0%)	13 (59.1%)	
Divorced or widowed	7 (35.0%)	9 (40.9%)	
Other	3 (15.0%)	0 (0.0%)	
Education			0.2
Grade 9–12	2 (10.0%)	6 (27.3%)	
College	10 (50.0%)	6 (27.3%)	
Post-graduate	8 (40.0%)	9 (40.9%)	
Other	0 (0.0%)	1 (4.6%)	
Duke comorbidity index	3.0 (1.1)	3.4 (1.3)	0.3
GDS	0.8 (1.0)	0.9 (1.4)	0.9
3MS	96.3 (4.3)	95.7 (3.1)	0.5
Trails A (s)	29.4 (9.9)	34.9 (10.5)	0.1
Trails B (s)	66.2 (32.0)	82.4 (49.0)	0.2
Height (in.)	66.2 (3.6)	67.2 (3.6)	0.4
Weight (pounds)	184.8 (39.9)	180.9 (27.8)	0.5
Fear of falling	9 (45.0%)	10 (45.5%)	0.9
Fall prior year	8 (40.0%)	3 (13.6%)	0.07
Instrumented walkway gait speed (m/s)	0.96 (0.15)	0.93 (0.17)	0.9

*SE* standard error, *GDS* Geriatric Depression Scale, *3MS* modified mini-mental status exam, *m/s* meters/second

There were no between arm differences in the fNIRS signal or gait speeds at baseline; those in the standard-plus arm had better alphabet task performance (Table 2). Mean values for fNIRS signal (both HbO and Hbr) and performance measures at all time points are provided in Supplemental Tables 1 and 2.

**Table 2:** Baseline functional near-infrared spectroscopy (fNIRS) and performance measures of older adults enrolled in a randomized clinical trial of a timing and coordination physical therapy intervention (*n* = 42)

Characteristic	Standard Plus	Standard	p-Value
	*N* = 20	*N* = 22	
	Mean (SE)	Mean (SE)	
Right-side HbO			
Even floor	0.79 (0.68)	1.08 (0.47)	0.7
Even floor alphabet	1.53 (0.53)	1.05 (0.45)	0.5
Uneven floor	1.88 (0.63)	1.74 (0.32)	0.8
Uneven floor alphabet	1.46 (0.58)	0.90 (0.34)	0.4
Left-side HbO			
Even floor	0.41 (0.58)	0.91 (0.45)	0.5
Even floor alphabet	1.21 (0.54)	1.17 (0.35)	0.9
Uneven floor	1.59 (0.59)	1.29 (0.37)	0.7
Uneven floor alphabet	1.04 (0.61)	0.90 (0.28)	0.8
Gait speed (m/s)			
Even floor	0.94 (0.04)	0.94 (0.03)	0.9
Even floor alphabet	0.86 (0.04)	0.83 (0.03)	0.5
Uneven floor	0.86 (0.04)	0.87 (0.03)	0.9
Uneven floor alphabet	0.78 (0.03)	0.77 (0.03)	0.8
Alphabet performance (letters/s)			
Even floor	0.65 (0.04)	0.54 (0.03)	0.03
Uneven floor	0.62 (0.03)	0.53 (0.04)	0.08

*SE* standard error, *HbO* oxygenated hemoglobin, *m/s* meters/second

The only significant pre- to post-intervention change in fNIRS was a decrease in HbO for the left PFC during dual-task walking on the even surface (even ABC) from baseline to 24 weeks in the standard-plus group (−1.05 ± 0.39; *p* = 0.02). The between arm differences in change of fNIRS HbO signal from baseline to follow-up visits were generally small and nonsignificant (Table 3, adjusted difference column). There were only two significant findings indicating a greater decrease in fNIRS HbO signal for both right and left PFC during uneven ABC at week 24 compared to baseline (right: −1.72 ± 0.80, *p* = 0.04; left: −1.79 ± 0.78, *p* = 0.03; Table 3) for the standard-plus compared to the standard arm. There was only one between intervention arm comparison that was significant for Hbr values (not shown in tables); this was for the right PFC during even ABC at 24 weeks indicating a greater decrease in Hbr in the standard-plus compared to the standard arm (between arm difference = −2.22, *p* = 0.003).

**Table 3:** Changes and mean differences between standard-plus (*n* = 20) and standard (*n* = 22) arms in prefrontal cortex oxygenated hemoglobin (HbO) by hemisphere, task, and visit

Brain hemisphere, Task	Time frame for change	Standard-plus Mean change (SE)	Standard Mean change (SE)	Adjusted difference (SE)^a^	Difference *p*-value^a^
Right, even floor	12 weeks	1.02 (0.67)	0.34 (0.62)	0.021 (0.75)	0.9
	24 weeks	1.07 (0.93)	0.068 (0.54)	0.28 (0.87)	0.8
	36 weeks	1.08 (0.92)	−0.35 (0.67)	0.30 (0.96)	0.8
Right even floor + alphabet	12 weeks	−0.77 (0.57)	0.49 (0.55)	−1.17 (0.67)	0.09
	24 weeks	−0.89 (0.55)	0.070 (0.63)	−1.01 (0.76)	0.2
	36 weeks	−0.60 (0.55)	−0.24 (0.75)	−1.00 (0.84)	0.2
Right, uneven floor	12 weeks	0.002 (0.86)	−0.80 (0.55)	0.46 (0.94)	0.6
	24 weeks	−0.74 (0.92)	−0.16 (0.49)	−0.28 (1.06)	0.8
	36 weeks	1.07 (0.68)	0.28 (1.14)	0.66 (1.16)	0.6
Right uneven floor + alphabet	12 weeks	−0.026 (0.48)	0.40 (0.61)	−0.29 (0.70)	0.7
	24 weeks	−1.25 (0.84)	0.57 (0.34)	−1.72 (0.80)	0.04
	36 weeks	0.72 (0.96)	−0.19 (0.83)	0.50 (0.88)	0.6
Left, even floor	12 weeks	0.78 (0.51)	0.092 (0.51)	−0.062 (0.65)	0.9
	24 weeks	0.81 (0.88)	0.24 (0.51)	−0.25 (0.74)	0.7
	36 weeks	1.05 (0.91)	−0.19 (0.70)	−0.38 (0.82)	0.6
Left even floor + alphabet	12 weeks	−0.51 (0.63)	0.24 (0.35)	−0.78 (0.61)	0.2
	24 weeks	−1.05 (0.39)*	−0.28 (0.44)	−0.87 (0.71)	0.2
	36 weeks	−0.80 (0.67)	−0.03 (0.47)	−1.00 (0.78)	0.2
Left, uneven floor	12 weeks	−0.15 (0.71)	−0.85 (0.58)	0.35 (0.84)	0.7
	24 weeks	−0.16 (0.83)	0.59 (0.69)	−0.61 (0.94)	0.5
	36 weeks	1.41 (0.80)	1.36 (1.26)	−0.46 (1.01)	0.7
Left uneven floor + alphabet	12 weeks	−0.045 (0.60)	0.24 (0.57)	−0.46 (0.68)	0.5
	24 weeks	−1.41 (0.76)	0.64 (0.57)	−1.79 (0.78)	0.03
	36 weeks	−0.14 (0.84)	0.01 (0.54)	−0.56 (0.86)	0.5

*SE* standard error

*p < 0.05 obtained using paired samples *t*-test

^a^Obtained using a linear mixed model

There were several significant changes from baseline for the channels D2S6 and D2S8 centered over the left dlPFC, but results did not indicate a consistent pattern (data not shown). D2S6 significantly increased in the standard-plus group from baseline to 12 weeks during even walking (0.72 ± 0.30, *p* = 0.03). D2S8 significantly decreased in the standard group from baseline to 24 weeks during even (−1.03 ± 0.45, *p* = 0.04) and even ABC (−0.96 ± 0.41, *p* = 0.04) and from baseline to 36 weeks during even (−1.43 ± 0.62, *p* = 0.04) walking. None of the between arm comparisons for change in D2S6 or D2S8 were significant (data not shown). While not significant, magnitudes of between-groups differences in these channel-specific results indicate that the greater decrease in left PFC HbO in the standard-plus group at 24 weeks during the uneven ABC task may be driven by D2S6 (upper left dlPFC) (−1.30 ± 0.67; *p* = 0.06) rather than D2S8 (lower left dlPFC; −0.65 ± 0.63; *p* = 0.3).

There were significant improvements in gait speed, particularly during even ABC and uneven ABC tasks, observed in both groups at week 12. These improvements persisted for the standard-plus group on ABC tasks at week 24 (Table 4). Rate of correct letter generation significantly improved at weeks 24 and 36 for both arms (Table 4). There were no significant between arm differences observed in the change in gait speed or letter generation over time (Table 4).

**Table 4:** Changes and mean differences between standard-plus (*n* = 20) and standard (*n* = 22) arms in gait speed and cognitive performance by task and visit

Brain hemisphere, Task	Time frame for change	Standard-plus Mean change (SE	Standard Mean change (SE	Adjusted difference (SE)^a^	Difference *p*-value^a^
Gait speed, even floor (m/s)	12 weeks	0.054 (0.022)*	0.046 (0.026)	0.004 (0.036)	0.9
	24 weeks	0.036 (0.020)	-0.018 (0.041)	0.059 (0.039)	0.1
	36 weeks	0.014 (0.036)	0.048 (0.029)	0.008 (0.041)	0.8
Gait speed, even floor + alphabet (m/s)	12 weeks	0.061 (0.029)*	0.059 (0.024)*	0.004 (0.035)	0.9
	24 weeks	0.055 (0.024)*	0.022 (0.032)	0.031 (0.038)	0.4
	36 weeks	0.059 (0.046)	0.052 (0.025)	0.041 (0.041)	0.3
Gait speed, uneven floor (m/s)	12 weeks	0.054 (0.022)*	0.047 (0.027)	0.005 (0.037)	0.9
	24 weeks	0.042 (0.022)	0.003 (0.042)	0.053 (0.039)	0.2
	36 weeks	0.032 (0.037)	0.057 (0.031)	0.036 (0.042)	0.4
Gait speed, uneven floor + alphabet (m/s)	12 weeks	0.070 (0.028)*	0.064 (0.027)*	0.003 (0.039)	0.9
	24 weeks	0.061 (0.026)*	0.033 (0.034)	0.027 (0.042)	0.5
	36 weeks	0.076 (0.037)	0.077 (0.039)	0.033 (0.045)	0.5
Alphabet performance, even floor (letters/s)	12 weeks	0.038 (0.022)	0.039 (0.027)	0.012 (0.034)	0.7
	24 weeks	0.048 (0.019)*	0.049 (0.017)*	0.001 (0.038)	0.9
	36 weeks	0.104 (0.038)*	0.112 (0.028)*	0.004 (0.042)	0.9
Alphabet performance, uneven floor (letters/s)	12 weeks	0.058 (0.023)*	0.058 (0.028)	0.41 (0.55)	0.5
	24 weeks	0.092 (0.031)*	0.086 (0.021)*	0.29 (0.65)	0.7
	36 weeks	0.124 (0.036)*	0.152 (0.036)*	0.37 (0.72)	0.6

*SE* standard error

^*^p < 0.05 obtained using paired samples *t*-test

^a^Obtained using a linear mixed model

## Discussion

In this ancillary study to a randomized controlled trial of a timing and coordination physical therapy program to improve walking speed in older adults, we found that PFC activation as measured by fNIRS generally did not change from pre- to post-intervention and activation did not significantly differ by intervention arm. The lack of consistent change in PFC activation was observed for both left and right hemispheres and for channels specific to the left dlPFC. There was a significant decrease in HbO for the right PFC during dual-task walking on the uneven surface from baseline to 24 weeks in the standard-plus group, consistent with our hypothesis that the training would improve gait automaticity and, therefore, result in decreased PFC activation during walking. However, this change is not supported by similar changes on other tasks or on this task at other time points which suggests that it is spurious. There were also no between arm differences observed for changes in performance for either gait speed or correct letter generation on any of the tasks. Both arms did experience an improved gait speed after the intervention which was most pronounced for the cognitive dual-task conditions. This is consistent with the findings from the main trial which found significant and clinically meaningful improvements in usual pace gait speed in both study arms but no difference between arms [19].

The extent of automatic versus attentional motor control may be assessed through fNIRS of the PFC during complex walking tasks. Walking in healthy adults is an automatic process with little reliance on the PFC. However, as automaticity of motor control diminishes in older adults due to impairments in the brain and other systems [5], activation of the PFC during walking tasks increases [9]. It’s possible that our results of an improvement in gait speed, particularly during dual-task conditions, coupled with no increase in PFC activation indicates an improved efficiency or automaticity in neural control of walking. Only one prior study was identified that assessed changes in PFC activation in older adults without neurologic disease during a motor skill training intervention [13]. In that study, older adults who completed 8 weeks of training on an integrated cognitive–motor dance video game compared to a balance and stretching program had significantly reduced PFC activation of left and right hemispheres after training, regardless of intervention arm. The dance game resulted in larger reductions of left PFC activation during the final 10 s of 30 s fast-paced treadmill walking compared to the balance program [13]. We only assessed overall activation during our overground walking tasks and not the temporal dynamics of the fNIRS signal. It is unknown whether changes in the temporal dynamics of the fNIRS signal have clinical significance.

Our study had several limitations, most notably we did not meet our enrollment goals due to COVID-19-related shutdowns of research operations. However, we were sufficiently close to our aim to enroll at least 50 participants. Also, there was no evidence for the presence of trends that did not reach significance due to insufficient sample size. Further, the large standard errors in our fNIRS estimates may suggest high variability in PFC response. This could have limited our ability to detect significance in changes, particularly if they occurred only in a subgroup of participants. Further research is needed to understand the variability of fNIRS signals and gait performance across older individuals. In addition, fNIRS measurements are limited to the cortical surface of the brain. As a result, we were unable to assess activation of additional relevant regions of the brain, such as the basal ganglia. We chose not to control multiple comparisons as this we considered to be an exploratory subsample analyses rather than a definitive trial and as a result, we were more concerned with missing a potential effect than with detecting one that wasn’t there. As our findings were largely negative, we did not believe this unduly affected the interpretation of our results. Finally, the sample included here was likely not representative of the general population of older adults in the US. Particularly notable was the high educational attainment of this sample with nearly 80% of the sample having a college degree or greater.

Our study had several notable strengths. Our study was a randomized clinical trial of a theory-based motor skill physical therapy intervention [5]. While the evidence for PFC involvement in dual-task walking in older adults is quite robust [9], few randomized clinical trials have assessed whether PFC activation during walking can be modified in older adults without overt neurological conditions [13, 14]. We assessed PFC function under both physically and cognitively demanding walking conditions to better capture the range of challenges experienced in daily mobility [34] and to distinguish between possible differences due to types of challenges. While generalizability of our sample based on demographics is limited, we did include a sample with a wide range of gait speeds and a representative burden of chronic conditions and falls. Finally, while we did not find significant between-groups differences, it is important to nonetheless document our findings from a rigorously conducted randomized trial.

In conclusion, we did not find that participation in a 12-week timing and coordination physical therapy intervention improved gait automaticity as measured by PFC activation from fNIRS. There was evidence for improvements in gait speed, particularly under cognitive dual-task conditions, which did not differ by intervention arm. The role of exercise interventions in improving automaticity of gait in older adults is unclear, and more direct interventions targeting neural control of mobility, such as inclusion of transcranial direct stimulation, may be needed.

### Supplementary Information

Below is the link to the electronic supplementary material.

Supplementary file1 (DOCX 24 KB)

## Data Availability

The datasets generated and analyzed during the current study are available from the corresponding author on reasonable request.
