# Supplementary material for: Prefrontal cortex activation while walking did not change but gait speed improved after a randomized physical therapy intervention
Source: Aging Clin Exp Res. 2024 Feb 17;36(1):43. doi: 10.1007/s40520-023-02666-7 (PMC10874329; doi:10.1007/s40520-023-02666-7)
Supplement: Supplementary file 1 — Supplementary file1 (DOCX 24 KB) [file 40520_2023_2666_MOESM1_ESM.docx]

Supplemental Table 1 – Functional near-infrared spectroscopy values for oxygenated (HbO) and deoxygenated (Hbr) hemoglobin changes by prefrontal cortex hemisphere under 4 walking task conditions compared to quiet standing. Results shown by intervention arm (Standard+, n=20; Standard, n=22) and by visit.

|  |  |  | baseline |  | 12-week |  | 24-week |  | 36-week |  |
| --- | --- | --- | --- | --- | --- | --- | --- | --- | --- | --- |
|  |  |  | Standard+ | Standard | Standard+ | Standard | Standard+ | Standard | Standard+ | Standard |
|  |  |  | Mean (SD) | Mean (SD) | Mean (SD) | Mean (SD) | Mean (SD) | Mean (SD) | Mean (SD) | Mean (SD) |
| HbO | right | Even | 0.79 (3.02) | 1.08 (2.18) | 1.33 (2.16) | 1.38 (2.48) | 1.16 (2.88) | 1.42 (1.44) | 0.63 (3.01) | 0.96 (2.24) |
|  |  | Even ABC | 1.53 (2.36) | 1.05 (2.11) | 0.58 (1.92) | 1.37 (2.29) | 0.33 (2.49) | 1.29 (1.60) | 0.33 (1.80) | 1.05 (2.68) |
|  |  | Uneven | 1.88 (2.83) | 1.74 (1.49) | 1.69 (3.63) | 1.04 (2.07) | 0.64 (3.60) | 1.60 (1.84) | 1.71 (2.94) | 2.04 (3.21) |
|  |  | Uneven ABC | 1.46 (2.61) | 0.90 (1.57) | 1.21 (2.18) | 1.15 (2.44) | -0.05 (2.76) | 1.81 (1.32) | 0.89 (1.42) | 0.91 (2.19) |
|  | left | Even | 0.41 (2.59) | 0.91 (2.11) | 0.87 (1.91) | 0.99 (1.90) | -0.03 (2.61) | 1.55 (1.67) | 0.24 (3.18) | 1.08 (2.05) |
|  |  | Even ABC | 1.21 (2.42) | 1.17 (1.63) | 0.60 (2.54) | 1.21 (2.21) | **-1.59 (3.07)** | 1.19 (1.73) | 0.18 (2.29) | 1.05 (2.19) |
|  |  | Uneven | 1.59 (2.64) | 1.29 (1.76) | 1.27 (2.25) | 0.69 (1.71) | 1.96 (2.66) | 2.00 (2.12) | 1.98 (3.58) | 2.46 (3.60) |
|  |  | Uneven ABC | 1.04 (2.73) | 0.90 (1.32) | 0.84 (2.44) | 1.17 (2.21) | 1.11 (2.10) | 1.78 (2.16) | 0.22 (1.27) | 1.18 (2.00) |
|  |  |  |  |  |  |  |  |  |  |  |
| Hbr | right | Even | 0.34 (2.28) | 0.82 (1.83) | 1.06 (2.78) | 0.86 (1.47) | -0.03 (2.61) | 0.21 (1.41) | 0.21 (2.33) | 0.66 (2.19) |
|  |  | Even ABC | -0.28 (1.86) | -0.03 (2.23) | -0.23 (2.41) | -0.31 (2.03) | -1.59 (3.07) | 0.62 (2.35) | -1.53 (1.85) | -0.19 (2.25) |
|  |  | Uneven | 1.88 (2.10) | 1.50 (2.89) | 1.36 (2.88) | 1.64 (2.16) | 1. 96 (2.66) | 1.26 (1.74) | 1.66 (2.38) | 2.09 (3.23) |
|  |  | Uneven ABC | 0.83 (2.02) | -0.27 (2.14) | -0.01 (4.09) | -0.10 (1.67) | 1.11 (2.10) | 0.10 (2.52) | -0.19 (1.89) | 1.37 (2.97) |
|  | left | Even | 0.48 (2.16) | 0.93 (1.91) | 1.01 (1.71) | 1.05 (1.33) | 0.85 (1.73) | 0.87 (1.84) | 1.07 (1.85) | 0.81 (1.58) |
|  |  | Even ABC | 0.23 (1.99) | 0.74 (1.76) | -0.23 (1.70) | -0.16 (2.35) | -0.49 (1.17) | 0.19 (1.78) | -0.94 (2.01) | -0.13 (1.68) |
|  |  | Uneven | 1.89 (1.80) | 1.10 (2.23) | 0.98 (3.08) | 0.85 (1.81) | 1.58 (1.95) | 1.98 (2.10) | 2.46 (2.16) | 2.19 (1.99) |
|  |  | Uneven ABC | 0.35 (2.47) | -0.22 (1.27) | -0.48 (3.02) | -0.16 (1.92) | 0.39 (1.49) | -0.20 (1.81) | -0.11 (2.16) | 0.28 (2.08) |

Bold indicates significant (p<0.05) difference from baseline

Supplemental Table 2 – Gait speed and alphabet task performance under 4 walking task conditions by intervention arm (Standard+, n=20; Standard, n=22) and by visit.

|  |  | Baseline |  | 12 week |  | 24 week |  | 36 week |  |
| --- | --- | --- | --- | --- | --- | --- | --- | --- | --- |
|  |  | Standard+ | Standard | Standard+ | Standard | Standard+ | Standard | Standard+ | Standard |
|  |  | Mean (SD) | Mean (SD) | Mean (SD) | Mean (SD) | Mean (SD) | Mean (SD) | Mean (SD) | Mean (SD) |
| Gait speed (meters/s) | Even | 0.94 (0.17) | 0.94 (0.15) | **1.01 (0.17)** | 0.99 (0.16) | 0.98 (0.20) | 0.95 (0.18) | 0.96 (0.20) | 0.96 (0.12) |
|  | Even ABC | 0.86 (0.16) | 0.83 (0.13) | **0.93 (0.19)** | **0.89 (0.16)** | **0.93 (0.20)** | 0.85 (0.17) | 0.95 (0.23) | 0.85 (0.10) |
|  | Uneven | 0.86 (0.16) | 0.87 (0.15) | **0.94 (0.17)** | 0.92 (0.17) | 0.91 (0.21) | 0.89 (0.17) | 0.92 (0.21) | 0.89 (0.13) |
|  | Uneven ABC | 0.78 (0.14) | 0.77 (0.12) | **0.86 (0.19)** | **0.84 (0.16)** | **0.86 (0.20)** | 0.81 (0.16) | 0.89 (0.21) | 0.82 (0.14) |
| Alphabet (letters/s) | Even ABC | 0.65 (0.16) | 0.54 (0.16) | 0.72 (0.13) | 0.61 (0.20) | **0.75 (0.12)** | **0.62 (0.19)** | **0.80 (0.18)** | **0.65 (0.20)** |
|  | Uneven ABC | 0.62 (0.14) | 0.53 (0.18) | **0.70 (0.13)** | 0.61 (0.19) | **0.75 (0.13)** | **0.63 (0.19)** | **0.76 (0.12)** | **0.68 (0.24)** |

Bold indicates significant (p<0.05) difference from baseline
